# Some Indications for the Use of Normal Saline Solution

**Published:** 1903-12

**Authors:** J. B. Morgan

**Affiliations:** Augusta, Ga.


### SOME INDICATIONS FOR THE USE OF NORMAL SALINE SOLUTION.*

By J. B. MORGAN, M. D.,

AUGUSTA, GA.

If it be true that the paramount duty of our profession is to save life, then the subject of this paper must be important, and I hope not uninteresting. My experience and observation in a wide range of emergency cases convinces me that we have at our command no more efficient and reliable agent for the saving of life than in the proper and prompt use of the normal saline solution.

I am perfectly aware of how extensively its benefits are known and its virtues invoked. While this is true, and serves as another illustration of the wonderful advances of modern medicine, it is not enough, for it is equally true that a great many good physicians do not use saline solution at all, and others employ it only in a few of the cases where it is so clearly indicated.

The frequent and extended use of normal salt solution since

* Read before the Georgia Medical Association, April, 1903.

1891 has satisfactorily demonstrated to all students of medicine its life-saving properties, and the fact that it is not now universally employed is not easily explainable.

I can understand why some general practitioners should fight shy of it on account of indefinable fear that the technique is essentially surgical; that the use of it might be attended with some known or unknown danger; that it involves cutting, and therefore, a review of anatomy, in order to find or avoid certain structures; but I should be loath to believe that any surgeon, from ignorance, indifference or laziness, would fail to give his patient the benefit of this agent in cases requiring its use.

Whatever the reasons may be which operate to deter some surgeons from the employment of this valuable life-saver, so well are its indications defined and its benefits understood, that a failure to resort to it will certainly bring regrets to the surgeon, with danger and perhaps death to his patient.

Neither time nor inclination will allow me to burden you with a review of cases in which I have found the use of saline solution of distinct and pronounced value in tiding patients over the effects of hemorrhage and shock, and of many other conditions, producing subnormal, intracardiac and intravascular pressure. The number would be in the hundreds, for normal salt solution is employod almost daily in our hospitals at Augusta. Each day, and each case, seems to increase its indications and widen its field of usefulness.

My experience with normal salt solution is confined almost exclusively to surgical cases, but our medical staff employ it quite as frequently as we do on the surgical side, and quite as successfully.

Whenever we encounter severe hemorrhage from accident, operation, post-pareum or other causes, we have learned to regard saline solution as a sheet-anchor to our patients.

The limits of this paper will not admit of a discussion of the pathology and etiology of shock, but, next to sepsis, shock is the cause of the greatest fatality following major operations.

In normal salt solution we have not only our most reliable agent in relieving, but our most effective means of preventing this condition by the free use of it before or during an operation where the advent of shock seems a probability.

Important as they are, the indications for normal salt solution are by no means limited to surgery.

In apparently desperate cases of lobar pneumonia, in which all of the signs point to a deficiency of water in the blood, normal saline solution injected beneath the skin has often turned the scale in favor of recovery.

In uremia, while diuresis does not seem to be materially increased, the effect of its use is good. The general condition of the patient improves, the heart's action becomes quieter, arterial pressure increased, the poison circulating in the blood is diluted and thereby rendered less noxious to the cells.

In infectious diseases, septic conditions and severe anemia, the results in cases reported are excellent, and clinical experience confirms these results. In addition to improved heart action and arterial pressure, the red blood-corpuscles multiply more rapidly and the circulating poison is diluted, diminished and freely eliminated, especially through the kidneys. This "washing of the blood" should be continued as long as fresh toxins develop.

In cholera, dysentery, severe diarrhea, or any disease where there is a decidedly diminished quantity of water in the blood, a liberal use of normal salt solution is indicated, and without its use the resources of the physician will not have been exhausted.

Normal salt solution corresponds in its specific gravity with the normal serum of the blood, and is prepared by dissolving 93 grains of common table salt in 33½ ounces of distilled water—approximately a heaped teaspoonful of salt to the quart of water.

Various modifications of this formula have been suggested, such as the addition of sugar, carbonate of soda and other ingredients, all intended to more nearly approximate the chemical formula of the blood; but clinical experience fails as yet to discover any improvement over the normal salt solution.

After the addition of the salt, the water should be sterilized by boiling. It seems to present no advantages to have the salt sterilized separately from the water, but the salt is first added, then the solution sterilized. Where quick work is not a factor, we can afford to prepare the solution in an ideal way, but under some circumstances salt directly from the table and any kind of water that is hot enough will do. The way in which the solution is to be used will largely determine its preparation.

Saline solution can be given by three methods: (a) injected directly into a blood-vessel of either artery or vein, (b) subcutaneously, and (c) by rectum.

All of these methods are good, and each has its advantages and advocates. Where prompt action is necessary, intravenous infusion is best. A vein in the operating wound, or any vein, can be selected, but the median basilic is the one usually chosen. It is rendered turgid and prominent by proximal compression, rapidly exposed, and incised transversely for the reception of the blunt-pointed cannula, or, what is quicker, a large, sharp hypodermic needle is thrust directly into the vein.

The solution should be allowed to fill the tube, and flow from the cannula or needle before it is pushed into the vein, to prevent the entrance of air. All aseptic precautions must be observed, and the solution not permitted to enter too fast, for fear of overwhelming a very weak and tired heart. The time should never be less than ten or fifteen minutes to the quart. The easiest and safest manner of administering saline infusion is by rectum.

A fountain syringe and rectal tube, or a large, soft rubber catheter, comprise the entire apparatus necessary. Elevate the foot of the table or bed, inject it high and hot, and the effect is prompt and positive. Where the emergency is not too great, or the rectum too irritable, this method is very effective, and it has none of the disadvantages of either intravenous or hypodermic injections. A pint or more of the solution may be used, and if the collapse is grave or the hemorrhage has been great, it may be repeated two or three times in the course of a day.

I have seen as much as two quarts given at one time in this way, and every drop taken up by the thirsty blood-vessels. Saline infusion is often given by hypodermoclysis, but is somewhat slow in its effects, the lymphatics not being especially prompt in allowing the diffusion of the solution from the connective tissues.

Still, in cases where the indications are not too urgent, or the bowel becomes intolerant, the subcutaneous infusion, by reason of its simplicity, is often employed; in medical cases it is probably the most desirable. There is not the slightest danger of throwing in the solution too fast by this method.

After the needle and skin have been sterilized, the needle is plunged into the subcutaneous tissue of the buttock, loin, scapula, or submammary regions, and the fountain syringe hung several feet above the bed, or, if the Davidson syringe is employed, the bulb slowly and regularly but firmly pressed. As the fluid runs in slowly, absorption will be greatly facilitated by gently manipulating the infiltrated area, or by placing hot towels over it. When the amount desired has been introduced, the needle is withdrawn and the small puncture in the skin closed with collodion.

If the condition of the patient is such that more than a pint must be given, the operation should be repeated in another region. Many instruments and apparatus, some rather complicated, have been devised for infusion in the veins and under the skin.

Some are good, none are necessary. Kelly's apparatus, with graduated bottle and force bulb in stopper, is probably the best.

Webster's little case is compact and convenient. A fountain or Davidson's syringe, a small piece of rubber tubing or a soft rubber catheter, and a large hypodermic needle constitute the entire necessary outfit for the intravenous or subcutaneous methods. Common and simple as they are, with their aid a perfectly effective apparatus can be prepared and used in a few minutes.

Dr. Dawbarn, of New York, was the first to call attention to the importance and value of having the saline solution at a higher temperature than that recommended by the text-books.

I have carefully observed the effects of the infusion in a variety of cases, at temperatures ranging from 100° to 120° F., and in every instance the higher the temperature the more rapid and decided was the effect on the heart and blood-vessels. The solution should be from 115° to 120° F., and maintained at that temperature by the application of some form of external heat to the vessel containing the salt water to be injected.

If a thermometer is not convenient, use the solution as hot as the hand can tolerate. This will be about 120° F. If it is injected at a temperature much above this point it may cause destruction of tissue. Since, however, globulin and serum albumin do not coagulate at less than about 160° F., it follows that we are well within safe limits at 120° F.

Whatever virtue normal salt solution may possess, it is undoubtedly enhanced by being used as hot as the protection of the tissues will permit. The marked effect of heat on the activity of all unstriped muscle is too well understood and appreciated to require argument here; it is necessary only to suggest that the muscular coat of blood-vessels is of the unstriped variety.

The quantity to be employed is dependent on the condition of the patient, from one to three pints being the usual variations, and one quart or liter about the average amount. Where infusion is indicated at all, less than one pint in case of adults, in my experience, is useless. I do not claim that salt solution is curative, or that it exerts marked beneficial action on general nutrition, but as a means of bridging our patients over some dangerous condition where death seems impending, it has few, if any equals. It can be employed by the general practitioner as readily as by the surgeon, and is as available at any home as at a well-equipped hospital.

